# Dichloridobis[2-(morpholin-4-yl)ethanamine-κ^2^ *N*,*N*′]cadmium

**DOI:** 10.1107/S1600536811003709

**Published:** 2011-02-02

**Authors:** Nura Suleiman Gwaram, Hamid Khaledi, Hapipah Mohd Ali

**Affiliations:** a Department of Chemistry, University of Malaya, 50603 Kuala Lumpur, Malaysia

## Abstract

In the title compound, [CdCl_2_(C_6_H_14_N_2_O)_2_], the Cl and Cd^II^ atoms are located on a twofold rotation axis and the Cd^II^ atom is octa­hedrally coordinated by two *N*,*N*′-bidentate 2-(morpholin-4-yl)ethanamine ligands and two *trans*-located Cl atoms. In the crystal, adjacent mol­ecules are linked by N—H⋯Cl and C—H⋯O hydrogen bonds into a three-dimensional structure. An intra­molecular C—H⋯Cl hydrogen bond is also observed.

## Related literature

For the structures of nickel(II) complexes with 4-(2-amino­eth­yl)morpholine (*L*), see: Chattopadhyay *et al.* (2005 ▶); Laskar *et al.* (2001 ▶). For the structures of other metal complexes with the ligand (*L*), see: Shi *et al.* (2006 ▶) and literature cited therein.
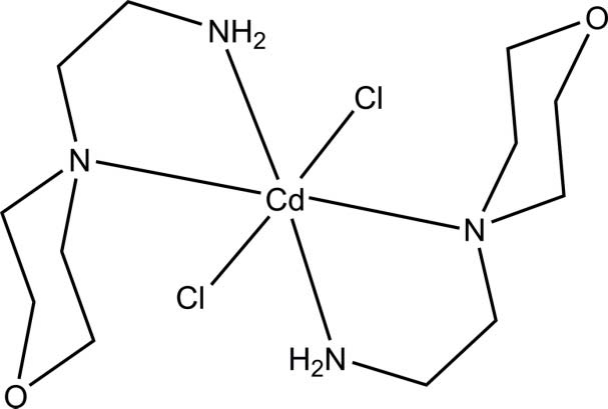


## Experimental

### 

#### Crystal data

[CdCl_2_(C_6_H_14_N_2_O)_2_]
*M* *_r_* = 443.68
Orthorhombic, 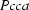

*a* = 19.6443 (2) Å
*b* = 10.6159 (1) Å
*c* = 8.3553 (1) Å
*V* = 1742.43 (3) Å^3^
*Z* = 4
Mo *K*α radiation
μ = 1.57 mm^−1^
*T* = 100 K
0.18 × 0.16 × 0.03 mm

#### Data collection

Bruker APEXII CCD diffractometer
Absorption correction: multi-scan (*SADABS*; Sheldrick, 1996 ▶) *T*_min_ = 0.765, *T*_max_ = 0.954
20511 measured reflections
2009 independent reflections
1619 reflections with *I* > 2σ(*I*)
*R*_int_ = 0.026

#### Refinement

*R*[*F* ^2^ > 2σ(*F* ^2^)] = 0.039
*wR*(*F* ^2^) = 0.078
*S* = 1.28
2009 reflections
103 parameters
2 restraints
H atoms treated by a mixture of independent and constrained refinement
Δρ_max_ = 1.00 e Å^−3^
Δρ_min_ = −1.06 e Å^−3^

### 

Data collection: *APEX2* (Bruker, 2007 ▶); cell refinement: *SAINT* (Bruker, 2007 ▶); data reduction: *SAINT*; program(s) used to solve structure: *SHELXS97* (Sheldrick, 2008 ▶); program(s) used to refine structure: *SHELXL97* (Sheldrick, 2008 ▶); molecular graphics: *X-SEED* (Barbour, 2001 ▶); software used to prepare material for publication: *SHELXL97* and *publCIF* (Westrip, 2010 ▶).

## Supplementary Material

Crystal structure: contains datablocks I, global. DOI: 10.1107/S1600536811003709/si2331sup1.cif

Structure factors: contains datablocks I. DOI: 10.1107/S1600536811003709/si2331Isup2.hkl

Additional supplementary materials:  crystallographic information; 3D view; checkCIF report

## Figures and Tables

**Table 1:** Selected bond lengths (Å)

Cd1—N2	2.290 (3)
Cd1—N1	2.537 (3)
Cd1—Cl2	2.6244 (13)
Cd1—Cl1	2.6414 (14)

**Table 2:** Hydrogen-bond geometry (Å, °)

*D*—H⋯*A*	*D*—H	H⋯*A*	*D*⋯*A*	*D*—H⋯*A*
N2—H2*C*⋯Cl2^i^	0.88 (3)	2.54 (3)	3.344 (3)	152 (4)
N2—H2*D*⋯Cl1^ii^	0.90 (3)	2.46 (3)	3.333 (3)	161 (4)
C1—H1*B*⋯Cl1	0.99	2.80	3.540 (4)	132
C5—H5*B*⋯O1^iii^	0.99	2.57	3.509 (5)	158

Symmetry codes: (i) 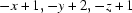; (ii) 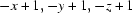; (iii) 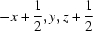.

**Fig. 1.**
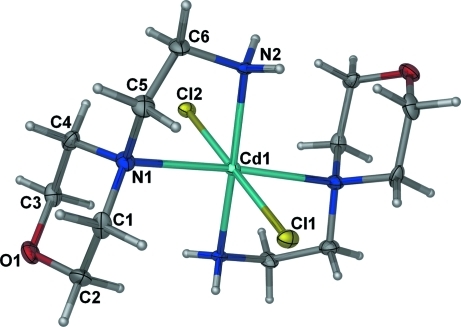
Thermal ellipsoid plot of the title compound at the 50% probability level. Unlabelled non-H atoms in the complex are related to labelled atoms by [1 - x, y, 1/2 - z].

## supplementary crystallographic information

### Comment

The title compound was obtained upon complexation of 4-(2-aminoethyl)morpholine with CdCl_2_. Similar to what was observed in the other metal complexes of 4-(2-aminoethyl)morpholine (Chattopadhyay *et al.*, 2005; Laskar *et al.*, 2001), the morpholine ring adopts a chair conformation and the amine acts as an *N,N'*-bidentate ligand to form a five-membered chelate ring with the metal center. Within the formed chelate ring, the Cd—N distances are considerably different from one another (Table 1). By contrast, the Pt—N bond lenghts in the square-planar complex of PtCl_2_ with the amine ligand (Shi *et al.*, 2006) are only slightly different [2.018 (6) and 2.075 (5) Å]. The Cd^II^ ion, placed on a 2-fold rotation axis, is six-coordinated by two of the amine ligands and two Cl atoms in a distorted octahedral geometry. The crystal structure is consolidated by intermolecular N—H···Cl and C—H···O and also intramolecular C—H···Cl hydrogen bonding interactions (Table 2).

### Experimental

A solution of cadmium(II) chloride (0.92 g, 5.0 mmol) in minimum amount of water was added to an ethanolic solution (50 ml) of 4-(2-aminoethyl)morpholine (1.30 g, 10 mmol). The resulting solution was refluxed for 30 min, then left at room temperature. The crystals of the title complex were obtained in a few days.

### Refinement

The C-bound hydrogen atoms were placed at calculated positions (C—H 0.99 Å) and were treated as riding on their parent atoms. The amine hydrogen atoms were located in a difference Fourier map and refined with a restrained N—H distance of 0.91 (3) Å. For all hydrogen atoms *U*iso(H) were set to 1.2 times *U*eq(carrier atom).

### Crystal data

**Table:**

[CdCl_2_(C_6_H_14_N_2_O)_2_]	*F*(000) = 904
*M**_r_* = 443.68	*D*_x_ = 1.691 Mg m^−^^3^
Orthorhombic, *P**c**c**a*	Mo *K*α radiation, λ = 0.71073 Å
Hall symbol: -P 2a 2ac	Cell parameters from 6671 reflections
*a* = 19.6443 (2) Å	θ = 3.3–30.4°
*b* = 10.6159 (1) Å	µ = 1.57 mm^−^^1^
*c* = 8.3553 (1) Å	*T* = 100 K
*V* = 1742.43 (3) Å^3^	Plate, colorless
*Z* = 4	0.18 × 0.16 × 0.03 mm

### Data collection

**Table:**

Bruker APEXII CCD diffractometer	2009 independent reflections
Radiation source: fine-focus sealed tube	1619 reflections with *I* > 2σ(*I*)
graphite	*R*_int_ = 0.026
φ and ω scans	θ_max_ = 27.5°, θ_min_ = 1.9°
Absorption correction: multi-scan (*SADABS*; Sheldrick, 1996)	*h* = −25→24
*T*_min_ = 0.765, *T*_max_ = 0.954	*k* = −13→13
20511 measured reflections	*l* = −10→10

### Refinement

**Table:**

Refinement on *F*^2^	Primary atom site location: structure-invariant direct methods
Least-squares matrix: full	Secondary atom site location: difference Fourier map
*R*[*F*^2^ > 2σ(*F*^2^)] = 0.039	Hydrogen site location: inferred from neighbouring sites
*wR*(*F*^2^) = 0.078	H atoms treated by a mixture of independent and constrained refinement
*S* = 1.28	*w* = 1/[σ^2^(*F*_o_^2^) + (0.*P*)^2^ + 9.8151*P*] where *P* = (*F*_o_^2^ + 2*F*_c_^2^)/3
2009 reflections	(Δ/σ)_max_ < 0.001
103 parameters	Δρ_max_ = 1.00 e Å^−^^3^
2 restraints	Δρ_min_ = −1.06 e Å^−^^3^

### Special details

**Table:**

Geometry. All e.s.d.'s (except the e.s.d. in the dihedral angle between two l.s. planes) are estimated using the full covariance matrix. The cell e.s.d.'s are taken into account individually in the estimation of e.s.d.'s in distances, angles and torsion angles; correlations between e.s.d.'s in cell parameters are only used when they are defined by crystal symmetry. An approximate (isotropic) treatment of cell e.s.d.'s is used for estimating e.s.d.'s involving l.s. planes.
Refinement. Refinement of *F*^2^ against ALL reflections. The weighted *R*-factor *wR* and goodness of fit *S* are based on *F*^2^, conventional *R*-factors *R* are based on *F*, with *F* set to zero for negative *F*^2^. The threshold expression of *F*^2^ > σ(*F*^2^) is used only for calculating *R*-factors(gt) *etc*. and is not relevant to the choice of reflections for refinement. *R*-factors based on *F*^2^ are statistically about twice as large as those based on *F*, and *R*- factors based on ALL data will be even larger.

### Fractional atomic coordinates and isotropic or equivalent isotropic displacement parameters (Å^2^)

**Table:**

	*x*	*y*	*z*	*U*_iso_*/*U*_eq_	
Cd1	0.5000	0.74719 (3)	0.2500	0.01253 (10)	
Cl1	0.5000	0.49837 (12)	0.2500	0.0181 (3)	
Cl2	0.5000	0.99440 (12)	0.2500	0.0173 (3)	
O1	0.31029 (15)	0.7816 (3)	−0.0516 (3)	0.0249 (7)	
N1	0.37113 (16)	0.7319 (3)	0.2563 (4)	0.0182 (6)	
N2	0.47563 (16)	0.7529 (3)	0.5179 (3)	0.0112 (6)	
H2C	0.497 (2)	0.815 (3)	0.566 (5)	0.013*	
H2D	0.491 (2)	0.682 (3)	0.565 (5)	0.013*	
C1	0.3397 (2)	0.6315 (4)	0.1558 (5)	0.0213 (9)	
H1A	0.2916	0.6197	0.1878	0.026*	
H1B	0.3640	0.5509	0.1736	0.026*	
C2	0.3431 (2)	0.6660 (4)	−0.0196 (5)	0.0256 (9)	
H2A	0.3913	0.6719	−0.0529	0.031*	
H2B	0.3213	0.5986	−0.0836	0.031*	
C3	0.3403 (2)	0.8793 (4)	0.0428 (5)	0.0212 (9)	
H3A	0.3166	0.9597	0.0206	0.025*	
H3B	0.3886	0.8895	0.0114	0.025*	
C4	0.3365 (2)	0.8516 (4)	0.2191 (5)	0.0213 (9)	
H4A	0.3583	0.9210	0.2796	0.026*	
H4B	0.2882	0.8465	0.2524	0.026*	
C5	0.3615 (2)	0.6927 (4)	0.4264 (5)	0.0217 (9)	
H5A	0.3753	0.6035	0.4383	0.026*	
H5B	0.3126	0.6990	0.4542	0.026*	
C6	0.4020 (2)	0.7721 (4)	0.5403 (5)	0.0210 (9)	
H6A	0.3909	0.8620	0.5230	0.025*	
H6B	0.3894	0.7501	0.6516	0.025*	

### Atomic displacement parameters (Å^2^)

**Table:**

	*U*^11^	*U*^22^	*U*^33^	*U*^12^	*U*^13^	*U*^23^
Cd1	0.01464 (18)	0.01259 (17)	0.01036 (17)	0.000	0.00092 (15)	0.000
Cl1	0.0252 (7)	0.0131 (5)	0.0161 (6)	0.000	−0.0003 (6)	0.000
Cl2	0.0234 (6)	0.0130 (5)	0.0155 (6)	0.000	−0.0011 (5)	0.000
O1	0.0217 (15)	0.0351 (17)	0.0178 (14)	0.0025 (13)	−0.0058 (12)	0.0038 (13)
N1	0.0172 (14)	0.0242 (17)	0.0133 (14)	−0.0006 (13)	0.0010 (13)	0.0038 (16)
N2	0.0181 (14)	0.0074 (13)	0.0082 (13)	0.0010 (12)	−0.0001 (11)	−0.0005 (12)
C1	0.0172 (19)	0.0103 (17)	0.036 (3)	−0.0043 (15)	−0.0047 (18)	0.0025 (18)
C2	0.023 (2)	0.028 (2)	0.026 (2)	−0.0008 (18)	−0.0047 (18)	−0.0127 (19)
C3	0.018 (2)	0.019 (2)	0.026 (2)	−0.0005 (16)	−0.0020 (17)	0.0088 (17)
C4	0.0158 (18)	0.0178 (19)	0.030 (2)	0.0017 (15)	−0.0001 (17)	−0.0073 (17)
C5	0.023 (2)	0.024 (2)	0.017 (2)	−0.0008 (17)	0.0024 (16)	0.0048 (17)
C6	0.019 (2)	0.032 (2)	0.0114 (17)	0.0013 (17)	0.0030 (15)	0.0007 (17)

### Geometric parameters (Å, °)

**Table:**

Cd1—N2	2.290 (3)	C1—H1A	0.9900
Cd1—N2^i^	2.290 (3)	C1—H1B	0.9900
Cd1—N1	2.537 (3)	C2—H2A	0.9900
Cd1—N1^i^	2.537 (3)	C2—H2B	0.9900
Cd1—Cl2	2.6244 (13)	C3—C4	1.503 (6)
Cd1—Cl1	2.6414 (14)	C3—H3A	0.9900
O1—C2	1.411 (5)	C3—H3B	0.9900
O1—C3	1.430 (5)	C4—H4A	0.9900
N1—C4	1.475 (5)	C4—H4B	0.9900
N1—C1	1.490 (5)	C5—C6	1.500 (6)
N1—C5	1.493 (5)	C5—H5A	0.9900
N2—C6	1.472 (5)	C5—H5B	0.9900
N2—H2C	0.88 (3)	C6—H6A	0.9900
N2—H2D	0.90 (3)	C6—H6B	0.9900
C1—C2	1.513 (6)		
			
N2—Cd1—N2^i^	176.95 (15)	C2—C1—H1B	109.5
N2—Cd1—N1	76.88 (11)	H1A—C1—H1B	108.1
N2^i^—Cd1—N1	103.32 (11)	O1—C2—C1	112.0 (3)
N2—Cd1—N1^i^	103.32 (11)	O1—C2—H2A	109.2
N2^i^—Cd1—N1^i^	76.88 (11)	C1—C2—H2A	109.2
N1—Cd1—N1^i^	172.65 (15)	O1—C2—H2B	109.2
N2—Cd1—Cl2	88.47 (8)	C1—C2—H2B	109.2
N2^i^—Cd1—Cl2	88.47 (8)	H2A—C2—H2B	107.9
N1—Cd1—Cl2	93.68 (8)	O1—C3—C4	112.2 (3)
N1^i^—Cd1—Cl2	93.68 (8)	O1—C3—H3A	109.2
N2—Cd1—Cl1	91.53 (8)	C4—C3—H3A	109.2
N2^i^—Cd1—Cl1	91.53 (8)	O1—C3—H3B	109.2
N1—Cd1—Cl1	86.32 (8)	C4—C3—H3B	109.2
N1^i^—Cd1—Cl1	86.32 (8)	H3A—C3—H3B	107.9
Cl2—Cd1—Cl1	180.0	N1—C4—C3	110.6 (3)
C2—O1—C3	109.8 (3)	N1—C4—H4A	109.5
C4—N1—C1	107.9 (3)	C3—C4—H4A	109.5
C4—N1—C5	112.5 (3)	N1—C4—H4B	109.5
C1—N1—C5	106.5 (3)	C3—C4—H4B	109.5
C4—N1—Cd1	113.6 (2)	H4A—C4—H4B	108.1
C1—N1—Cd1	116.6 (2)	N1—C5—C6	112.4 (3)
C5—N1—Cd1	99.4 (2)	N1—C5—H5A	109.1
C6—N2—Cd1	109.5 (2)	C6—C5—H5A	109.1
C6—N2—H2C	108 (3)	N1—C5—H5B	109.1
Cd1—N2—H2C	111 (3)	C6—C5—H5B	109.1
C6—N2—H2D	113 (3)	H5A—C5—H5B	107.9
Cd1—N2—H2D	110 (3)	N2—C6—C5	111.3 (3)
H2C—N2—H2D	106 (4)	N2—C6—H6A	109.4
N1—C1—C2	110.7 (3)	C5—C6—H6A	109.4
N1—C1—H1A	109.5	N2—C6—H6B	109.4
C2—C1—H1A	109.5	C5—C6—H6B	109.4
N1—C1—H1B	109.5	H6A—C6—H6B	108.0

Symmetry codes: (i) −*x*+1, *y*, −*z*+1/2.

### Hydrogen-bond geometry (Å, °)

**Table:**

*D*—H···*A*	*D*—H	H···*A*	*D*···*A*	*D*—H···*A*
N2—H2C···Cl2^ii^	0.88 (3)	2.54 (3)	3.344 (3)	152 (4)
N2—H2D···Cl1^iii^	0.90 (3)	2.46 (3)	3.333 (3)	161 (4)
C1—H1B···Cl1	0.99	2.80	3.540 (4)	132
C5—H5B···O1^iv^	0.99	2.57	3.509 (5)	158

Symmetry codes: (ii) −*x*+1, −*y*+2, −*z*+1; (iii) −*x*+1, −*y*+1, −*z*+1; (iv) −*x*+1/2, *y*, *z*+1/2.
